# FXR agonist obeticholic acid induces liver growth but exacerbates biliary injury in rats with obstructive cholestasis

**DOI:** 10.1038/s41598-018-33070-1

**Published:** 2018-11-08

**Authors:** Rowan F. van Golen, Pim B. Olthof, Daniël A. Lionarons, Megan J. Reiniers, Lindy K. Alles, Zehra Uz, Lianne de Haan, Bulent Ergin, Dirk R. de Waart, Adrie Maas, Joanne Verheij, Peter L. Jansen, Steven W. Olde Damink, Frank G. Schaap, Thomas M. van Gulik, Michal Heger

**Affiliations:** 1 Department of Experimental Surgery, Academic Medical Center, University of Amsterdam, Amsterdam, The Netherlands; 2 Tytgat Institute for Liver and Intestinal Research, Academic Medical Center, University of Amsterdam, Amsterdam, The Netherlands; 3 Oncogene Biology Laboratory, The Francis Crick Institute and University College London, London, United Kingdom; 4 Department of Pathology, Academic Medical Center, University of Amsterdam, Amsterdam, The Netherlands; 5 Department of Surgery, NUTRIM School of Nutrition and Translational Research in Metabolism, Maastricht University, Maastricht, The Netherlands

## Abstract

Cholestasis impairs liver regeneration following partial liver resection (PHx). Bile acid receptor farnesoid X-receptor (FXR) is a key mediator of liver regeneration. The effects of FXR agonist obeticholic acid (OCA) on liver (re)growth were therefore studied in cholestatic rats. Animals underwent sham surgery or reversible bile duct ligation (rBDL). PHx with concurrent internal biliary drainage was performed 7 days after rBDL. Animals were untreated or received OCA (10 mg/kg/day) per oral gavage from rBDL until sacrifice. After 7 days of OCA treatment, dry liver weight increased in the rBDL + OCA group, indicating OCA-mediated liver growth. Enhanced proliferation in the rBDL + OCA group prior to PHx concurred with a rise in Ki67-positive hepatocytes, elevated hepatic *Ccnd1* and *Cdc25b* expression, and an induction of intestinal fibroblast growth factor 15 expression. Liver regrowth after PHx was initially stagnant in the rBDL + OCA group, possibly due to hepatomegaly prior to PHx. OCA increased hepatobiliary injury markers during BDL, which was accompanied by upregulation of the bile salt export pump. There were no differences in histological liver injury. In conclusion, OCA induces liver growth in cholestatic rats prior to PHx but exacerbates biliary injury during cholestasis, likely by forced pumping of bile acids into an obstructed biliary tree.

## Introduction

In surgical practice, the ability of the liver to regenerate is exploited to enable safe liver resection and transplantation. Initiation, propagation, and termination of liver regeneration after partial hepatectomy (PHx) are regulated by an elaborate cytokine-, growth factor-, and metabolic signaling network^1^. Whereas healthy livers regenerate proficiently, liver regeneration is impaired in patients with parenchymal pathology caused by, e.g., chemotherapy, steatosis, or cholestasis^2,3^.

The risks of performing major liver resection in patients with obstructive cholestasis were recognized decades ago and have shaped the current surgical management of jaundiced patients with a biliary malignancy. The poor regenerative capacity of cholestatic livers has spawned invasive procedures such as preoperative biliary drainage and portal vein embolization (PVE) to respectively relieve cholestasis and increase future remnant liver size prior to resection. Despite the successful implementation of these interventions, surgery for perihilar cholangiocarcinoma, which is the primary cause of cholestasis in patients scheduled for liver surgery, remains associated with a 90-day mortality rate of up to 14%^4^. An additional group of patients is excluded from surgery when these preoperative interventions fail to sufficiently increase liver size and/or function.

Novel drugs that activate bile acid (BA) receptors such as FXR^5^ may enable pharmacological control over liver growth. The rationale for using these compounds to enhance liver regeneration is based on animal studies showing that enterohepatic BA cycling with activation of intestinal and hepatic BA receptors such as Fxr and Tgr5 are crucial for normal progression of liver regeneration after PHx^6–8^. The importance of intestinal BA signaling for efficient liver regeneration likely relates to the Fxr-dependent production of mitogenic fibroblast growth factor 19 (FGF19, Fgf15 in rodents) by ileal enterocytes^9,10^. The translational aspects of these findings were apparent from clinical studies indicating that post-operative external biliary drainage suppresses liver regeneration after major hepatectomy^11^.

The recent clinical evaluation of the FXR agonist obeticholic acid (OCA) for treatment of primary biliary cirrhosis^12^ has generated promising results that led to marketing approval of OCA for treating patients non-responsive to first line treatment with UDCA. In rabbits, we recently showed that OCA accelerates hypertrophy of the non-embolized liver segments after PVE^13^. However, OCA has not yet been tested in the context of human liver regeneration or in preclinical studies on the regeneration of pre-damaged livers after PHx. Using a rat model of reversible obstructive cholestasis and PHx, we show that OCA treatment triggers ileal *Fgf15* expression and induces liver growth prior to resection. Nevertheless, liver regeneration after PHx was reduced in OCA-treated rats, possibly due to the expansion of liver size prior to PHx.

## Materials and Methods

### Animals

Male Wistar rats (275–325g, ±8 weeks old) were purchased from Harlan (Horst, the Netherlands), acclimated for one week, and housed under standardized laboratory conditions^14^. Prior to surgery, animals received 0.025 mg/kg of buprenorphine subcutaneously as analgesia. Anesthesia was induced and maintained with a mixture of O_2_ and isoflurane (Forene, Abbott Laboratories, Chicago, IL)^14^. The body temperature was maintained at 37.0 ± 0.2 °C with a heating mat and heating lamp. The experimental protocols were approved by the Institutional Review Board (‘dierexperimentencommissie’, BEX101653) and were performed in accordance with international and institutional guidelines.

### Experimental design

After the acclimatization period, rats were either maintained on a regular chow diet or were switched to a methionine and choline-deficient (MCD) diet (Harlan Teklad, Madison, WI) for 3 weeks to induce moderate hepatic steatosis without causing liver inflammation^14^. The MCD diet was chosen over of other dietary steatosis models because of extensive experience with the model in our group, to maintain comparability to previous studies, and due to reproducibility issues with high-fat diets in rats^15^. At the start of the second diet week, the rats either underwent sham surgery or were subjected to reversible bile duct ligation (rBDL). For rBDL, the extrahepatic bile duct was cannulated with a polyethylene catheter connected to Silastic Tubing with a closed distal tip^16^. Sham-operated animals underwent abdominal surgery, including bile duct mobilization, but without bile duct ligation. rBDL rats were left untreated or received a daily oral gavage of the FXR agonist OCA (10 mg/kg in 0.5% methylcellulose, 1.5 mL per 300 g body weight) between 7:30 and 9:00 AM. OCA was provided by Intercept Pharmaceuticals (New York, NY).

Seven days after BDL or sham operation, rats were subjected to 70% PHx^17^. In rBDL rats, bile flow towards the intestines was restored directly prior to PHx by removing the closed cannula tip and inserting the cannula into the duodenum^16^. Animals in all treatment groups were put on regular chow after PHx. OCA treatment was continued after PHx until sacrifice. The study consisted of five experimental groups: control (sham), steatosis (MCD), cholestasis (rBDL), combined steatosis and cholestasis (rBDL + MCD), and cholestasis + OCA (rBDL + OCA). The study design is depicted in Fig. S1. The power calculation is included in the Supplemental Information.

At baseline (i.e., directly before PHx) and one to five days after PHx, animals were euthanized under isoflurane anesthesia by exsanguination. Blood samples were collected in heparin-anticoagulated vacutainers (BD, Franklin Lakes, NJ). In rBDL animals sacrificed at baseline, bile was aspirated with a syringe from the dilated extrahepatic bile duct, weighed, and stored at −80 °C. The liver and ileum were excised, weighed, and either fixed in formalin solution, snap-frozen in liquid nitrogen, or collected in RNAlater (Qiagen, Venlo, the Netherlands). Liver regrowth was calculated from the regenerated liver mass and was expressed as percentage of total liver mass prior to PHx. The projected remnant liver mass was calculated using the weight of the resected liver segments, which represent 70% of the total liver mass. Dry liver weights were measured to correct for hepatic water content^18^.

### Histology

Liver sections were stained with hematoxylin and eosin (H&E), picrosirius red, or the proliferation marker Ki67^14,17^. Semi-quantitative analysis of liver histology (Table S1) was performed by a hepatopathologist (JV) blinded to the experimental groups. Ki67-positive hepatocyte nuclei were manually counted by two observers (RFG and PBO) in four randomly-selected, non-overlapping microscopic fields at 200× magnification.

### Quantitative real-time polymerase chain reaction

RNA isolation and gene expression analysis were performed as described^19^. Liver samples were homogenized with a MagNA Lyser and RNA was extracted with the High Pure RNA Tissue Kit (Roche, Basel, Switzerland). One μg of RNA was reverse transcribed to cDNA using the SensiFAST cDNA Synthesis Kit (Bioline, London, UK). QRT-PCR was performed on a LightCycler 480 (Roche) using SensiFAST SYBR No-ROX mix (Bioline). Fluorescence data were processed and analyzed using LinRegPCR software and normalized to *Hprt* for ileum samples, and the geometric mean of *Ubc* and *B2m* for liver samples. These genes proved most stable (data not shown). Primers were designed using NCBI Primer Blast to span an intron or exon-exon junction (Table S2). Melting curve analysis and agarose gel electrophoresis were used to validate primer specificity.

### High performance liquid chromatography analysis of bile acid pool composition

Bile acids were separated and quantified in 20 μL of bile of plasma or 100 mg of liver tissue by reverse-phase high-performance liquid chromatography as described in detail in^14^. Biliary bile acid concentrations were multiplied by the total volume of bile to calculate biliary bile acid output.

### Statistical analysis

Statistical analysis was performed with GraphPad Prism (GraphPad Software, La Jolla, CA) abiding by a significance level of 0.05. Power calculations were performed using Piface software (https://homepage.divms.uiowa.edu/~rlenth/Power/). Numerical data were analyzed using a Student’s t-test or one-way ANOVA with Tukey’s post-hoc correction. Histological scoring was analyzed using Chi-square tests. Correlations were tested using Pearson product-moment correlation coefficient.

## Results

### Obstructive cholestasis impairs liver regeneration following partial hepatectomy

Liver regeneration is often studied in animals with healthy livers, whereas livers of surgical patients are often affected by, e.g., chemotherapy, steatosis, or cholestasis. As these conditions potentially impair liver regeneration, liver regeneration after PHx was first studied under conditions of parenchymal pathology. Pilot experiments showed that obstructive cholestasis following rBDL considerably impairs liver regeneration after PHx, whereas the effects of simple steatosis or combined cholestasis and steatosis were less pronounced or even absent (Fig. S2). Based on these data, the remainder of the experiments focused on improving defective liver regeneration in rBDL animals. As parameters for liver function such as prothrombin time and antithrombin-III activity were not affected in rBDL animals, both prior to and after PHx (Fig. S10), these parameters were not measured in the remainder of the study.

### Obeticholic acid induces liver growth in cholestatic rats prior to liver resection

The diminished hepatic regenerative potential of rats subjected to rBDL could result from perturbed perioperative intestinal Fxr signaling caused by changes in luminal BA delivery and/or from toxicity related to intrahepatic BA accumulation. It was hypothesized that impaired liver regeneration in rBDL rats could be ameliorated by restoring intestinal Fxr signaling through oral administration of the FXR agonist OCA.

**Figure 1:**
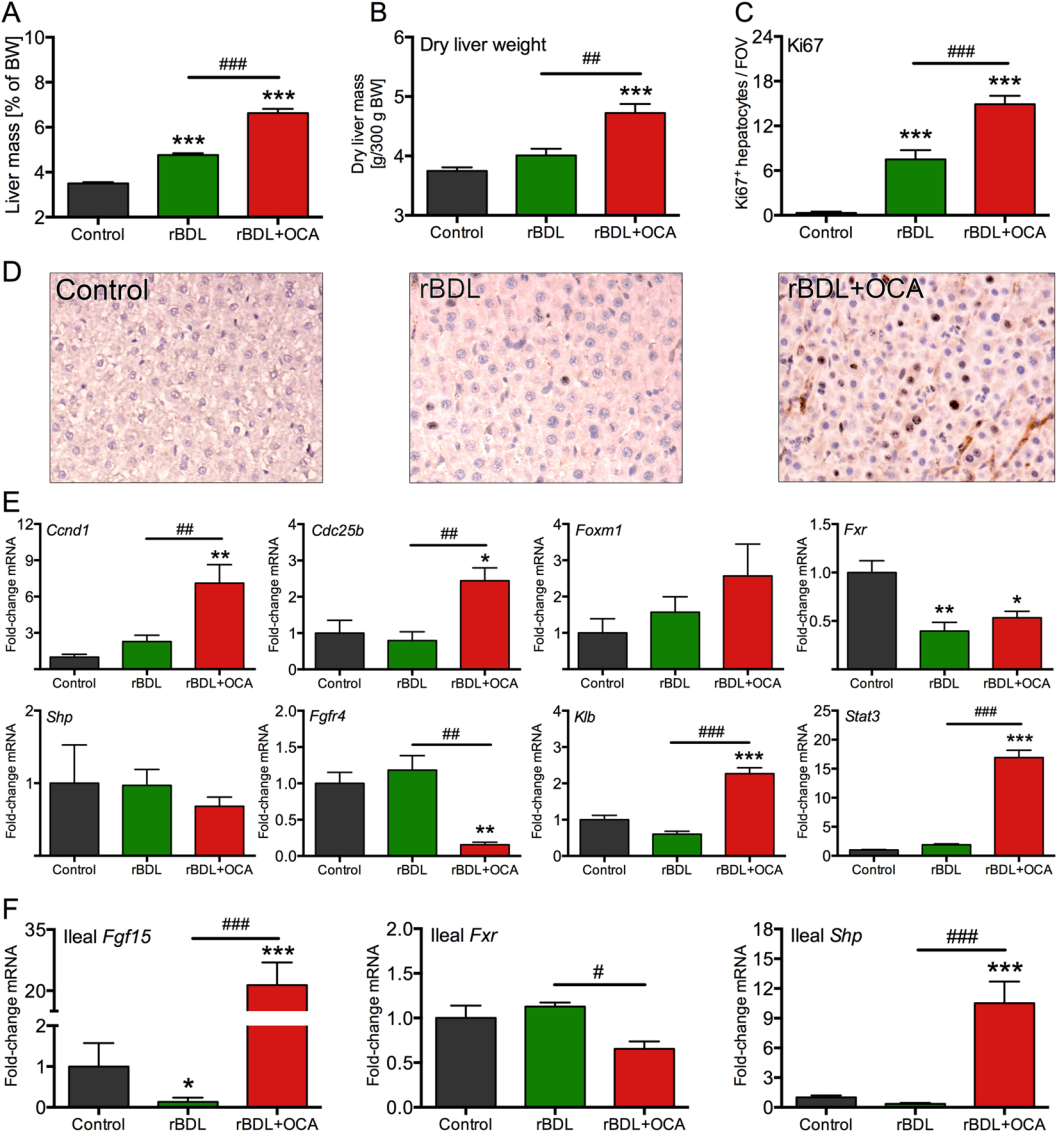
Obeticholic acid induces hepatocyte proliferation and hepatomegaly in cholestatic rats prior to liver resection. (**A**) Shows the total liver weight of controls (sham surgery) and after 7 days of rBDL with or without OCA treatment. Liver weight is expressed as percentage of body weight (**A**) or as dry weight per 300 g body weight (**B**,**C**) shows quantitative assessment of Ki67 positivity and (**D**) includes representative Ki67-stained liver sections. (**E**,**F**) Show expression of proliferation- and Fxr-related genes in liver or ileum, respectively. Levels are expressed relative to mean expression in the control rats. Abbreviations: BW = body weight; FOV = field of view; OCA = obeticholic acid; rBDL = reversible bile duct ligation (cholestasis). *Indicates p < 0.05, **indicates p < 0.01, and ***indicates p < 0.001, all versus the control group. ^#^Indicates p < 0.05, ^##^indicates p < 0.01, and ^###^indicates p < 0.001, all between the experimental groups indicated by the solid line. *N* = 4–6 per group per time point.

Gross liver mass prior to PHx was higher in both rBDL groups compared to the control group (Fig. 1A). After correcting for hepatic water content (e.g., due to inflammatory edema or volumetric expansion of the biliary tree^20^), increased liver mass was only observed in OCA-treated animals (Fig. 1B), confirming that rBDL-induced cholestasis was associated with inflammation^14^ and indicating that OCA stimulated liver growth under these conditions. Accordingly, hepatocyte proliferation, measured by Ki67 staining and expression of cell cycle markers *Ccnd1* (encoding cyclin D1) and *Cdc25b* (encoding M-phase inducer phosphatase 2), was more pronounced in the rBDL + OCA group compared to untreated rBDL or non-cholestatic animals (Fig. 1C–E). Both Ki67 staining and *Ccnd1* expression also correlated positively with dry liver mass (Fig. S3). As hepatocytes in native livers are in a quiescent state (Fig. 1C,D), these results suggest that proliferation in the rBDL groups is part of a homeostatic response aimed to repair cholestatic liver damage. However, liver hyperplasia did not occur during the seven-day rBDL period in the absence of OCA treatment as judged by the unaltered dry liver mass, despite the endogenous proliferative signals elicited by rBDL. A mutual effect of OCA treatment and cholestasis on liver size is also evident from the finding that liver growth did not occur in healthy rats treated with OCA for seven days (Fig. S4).

Animals in the rBDL group lost 1.7% of their body weight during 7 days of cholestasis, whereas the body weight of rats in the rBDL + OCA group decreased by 12.8% (Fig. S5). This 11.1% difference does not fully account for the 17.8% increase in dry liver mass observed between the rBDL an rBDL + OCA groups (Fig. 1B). In fact, it was previously shown that rat liver mass deteriorates considerably faster than body weight under conditions of poor nutrient intake^21^, implying that the observed effect of OCA on liver mass possibly underestimates the actual effect of OCA.

OCA-induced hepatocyte proliferation reportedly proceeds via a direct (hepatic) pathway and/or an indirect intestinal pathway. The direct pathway is mediated by the hepatocellular Fxr target gene *Foxm1b*^22^, which regulates cell cycle progression and is indispensable for effective liver regeneration after PHx^23^. The indirect pathway involves intestinal Fxr-linked production of mitogenic Fgf15 and signaling via its hepatic receptor complex composed of Fgfr4 and *β*klotho (encoded by *Klb*)^9,10,24^. It should be noted that there is no consensus on whether the indirect pathway also increases hepatic *Foxm1b* expression^10,22^.

Hepatic expression levels of *Fxr* and *Foxm1* were comparable between both rBDL groups (Fig. 1E). Accordingly, the direct pathway was most likely not responsible for the proliferative signaling induced by OCA. In contrast, ileal *Fgf15* transcription was ~20-fold upregulated in rBDL rats receiving OCA, which concurred with marked upregulation of the ileal Fxr target gene *Shp* (Fig. 1F). Hepatic *Fgf15* transcripts were not detectable in rBDL groups (data not shown). Thus, OCA treatment led to activation of intestinal Fxr, with a minor downregulation of ileal *Fxr* itself. Ileal *Fgf15* expression was strongly suppressed in untreated rBDL animals (Fig. 1F), likely owing to the failed delivery of ileal Fxr-activating ligands (i.e., BAs) after rBDL^6^. Fgf15 binds to its cognate receptor Fgfr4 on hepatocytes that, in the presence of βKlotho, leads to proliferative signaling through activation of the Stat3 pathway^10,24^. Although *Fgfr4* was downregulated at the transcript level, *Klb* and *Stat3* were considerably upregulated (Fig. 1E), suggesting that signaling through Fgfr4 was still effective. βKlotho could mediate this effect by stabilizing the Fgfr4 protein independent of Fgfr4 mRNA levels, as was shown *in vitro* using recombinant FGF19^25^. Accordingly, *Fgfr4* transcript levels were negatively correlated to dry liver mass, while *Stat3* transcript levels exhibited a strong positive correlation (Fig. S3). These findings support the involvement of the Fxr/Fgf15 axis in liver growth in OCA-treated rBDL rats.

### Liver regrowth after partial hepatectomy is reduced in post-cholestatic rats receiving obeticholic acid

**Figure 2:**
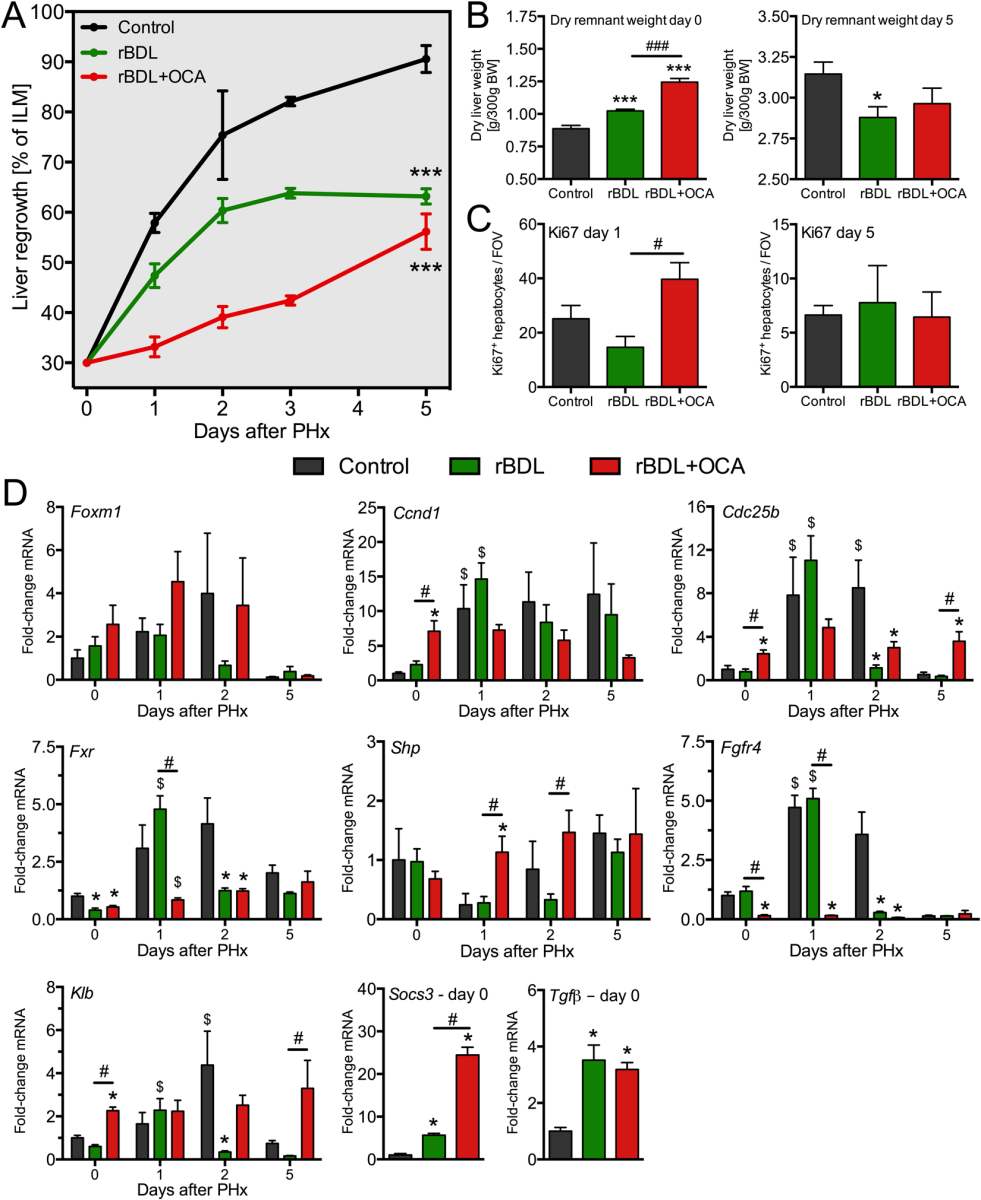
Liver regrowth is reduced in post-cholestatic rats treated with obeticholic acid. (**A**) Shows liver regrowth after partial hepatectomy (PHx) in healthy rats (black line), in untreated post-cholestatic rats (rBDL, green line), and rBDL rats receiving obeticholic acid (OCA, red line). Shown in (**B**) are the dry weights over the remnant liver immediately (left) and five days after PHx (right). (**C**) Shows the number of proliferating hepatocytes on day 1 (left) and day 5 (right) after PHx. (**D**) Presents the hepatic expression of various proliferation and Fxr-related genes. Levels are expressed relative to mean expression in the control rats at day 0. Abbreviations: BW = body weight; rBDL = reversible bile duct ligation (cholestasis); OCA = obeticholic acid. *Indicates p < 0.05 versus the control group. ^#^Signifies p < 0.05 between the experimental groups indicated by the solid line. ^$^Indicates p < 0.05 versus baseline (day 0) within an experimental group. *N* = 4–6 per group per time point.

After establishing that OCA increases the size of cholestatic livers, it was investigated whether OCA also accelerates liver regeneration following 70% PHx. Unexpectedly, liver regrowth was slower in OCA-treated rBDL animals compared to untreated rBDL and control rats (Fig. 2A). There are at least two explanations for this unexpected finding. First, *Fgfr4* expression was downregulated on the day of PHx (Fig. 1E). The slower recovery of liver mass in the OCA group could therefore result from reduced mitogenic Fgf15 signaling, although it should be noted that *Klb* expression remained constant. The finding that *Fgfr4* transcript levels were elevated on day 1 after PHx in control and untreated rBDL animals supports this line of thought (Fig. 2D, discussed below). Moreover, the early induction of *Fgfr4* and *Klb* following PHx in untreated rBDL rats is followed by a sharp decline in *Fgfr4* and *Klb* mRNA levels on day 2 post-PHx (Fig. 2D), which coincides with the cessation of liver regrowth in this group. Second, the dry weight of the remnant liver at the time of PHx was already higher in the OCA group than in the other two groups (Fig. 2B). As the rate of liver regeneration is proportional to the amount of liver removed, and thus inversely related to the size of the remnant liver^26^, the reduced regrowth rate after PHx in OCA-treated animals may have resulted from the expansion of liver size before resection. In line with this premise, the liver-to-body-weight ratio of rBDL + OCA rats was similar to control animals five days after PHx (Fig. 2B). This ratio remained lower in the untreated rBDL group relative to controls.

To closer investigate these findings, the expression of Fxr-related genes and proliferation parameters were monitored for five days after PHx. Apart from *Fgfr4*, hepatic *Fxr* was markedly upregulated on day one after PHx in controls and untreated rBDL rats (Fig. 2D). The induction of *Ccnd1* and *Cdc25b* concurrent with increased Ki67-positive hepatocytes (Fig. 2C,D) in these groups supports previous notions that an ileal Fxr-Fgf15-Fgfr4 signaling axis is required for liver regeneration^6,9^. Accordingly, the stagnating regrowth in untreated rBDL animals on day two after PHx was paralleled by a loss of *Fxr* and *Fgfr4* expression. This finding could not only explain the exhaustion of regeneration in post-cholestatic livers, but also strengthens the rationale for targeting this pathway to enhance liver regeneration.

In line with the strong pre-PHx induction of intestinal *Fgf15* expression (Fig. 1F), hepatic *Fgfr4* expression remained low in OCA-treated animals during the regrowth phase. Effects of OCA on hepatic *Fxr* were most pronounced during the first two days after Phx, as indicated by high transcript levels of the *Fxr* target *Shp* in the liver relative to untreated rBDL and control rats. The latter is supported by the slight decrease in liver *Fxr* mRNA on day 1 post-PHx. Despite the successful induction of hepatic Fxr targets by OCA, hepatic *Foxm1b* expression did not change during liver regeneration in any group (Fig. 2D).

In contrast to the regrowth kinetics, the number of Ki67-positive hepatocytes was highest in the OCA group on day one after PHx (Figs 2C and S6), indicating increased proliferation. This discrepancy may stem from the OCA-induced increase in liver mass prior to PHx. The induction of proliferative signaling by OCA before resection may have caused the peak in Ki67 staining observed on day one after PHx, while the loss of *Fgfr4* expression and induction of signals for termination of liver regeneration such as transforming growth factor beta (*Tgfβ*) and suppressor of cytokine signaling 3 (*Socs3*) (Fig. 2D) could account for the delayed recovery of initial liver mass in the OCA group.

### OCA aggravates biliary injury during obstructive cholestasis by modulating bile acid transport

#### Cholestasis

**Figure 3:**
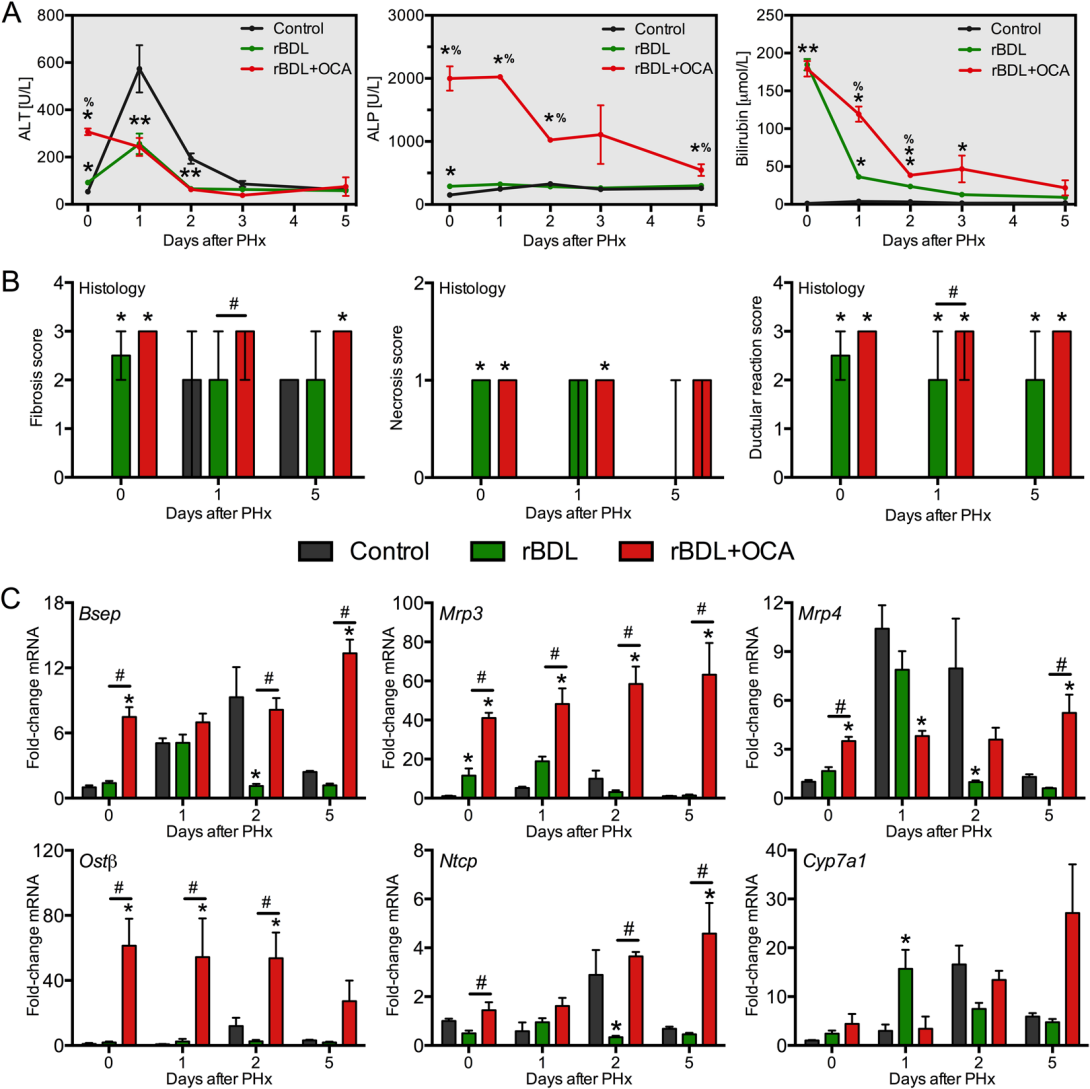
Obeticholic acid aggravates cholestatic injury through Bsep-mediated bile acid export. (**A**) Shows serum hepatobiliary injury markers, measured before (t = 0) and after partial hepatectomy (PHx) in controls rats (black line), and (post)cholestatic rats left untreated (green line) or receiving obeticholic acid (OCA, red line). (**B**) Shows histologic scoring of H&E-stained liver sections shown as mean score ± range. Apart from fibrosis from post-surgery day 1 onwards, no histological anomalies were observed in control rats. The scoring system and representative images are included as Supplementary Information. (**C**) Shows expression of genes related to hepatocyte BA homeostasis. Levels are expressed relative to mean expression in the control rats at day 0. Abbreviations: ALP = alkaline phosphatase; ALT = alanine aminotransferase; rBDL = reversible bile duct ligation (cholestasis); OCA = obeticholic acid. *Indicates p < 0.05 versus the control group and ^#^signifies p < 0.05 versus the experimental group indicated by the solid line. *N* = 4–6 per group per time point.

As several reports have found that genetic deletion of Fxr reduces BDL-induced liver injury^27^, potential downsides of OCA treatment during rBDL were also studied. Prior to PHx, alanine aminotransferase (ALT) and alkaline phosphatase (ALP) levels were considerably higher in rBDL + OCA rats than in the other two groups, indicating aggravated hepatobiliary injury (Fig. 3A). Plasma gamma glutamyltransferase levels followed the same dynamics as ALP (Fig. S7). Histological analysis revealed a similar extent of hepatocellular necrosis and fibrotic changes in the rBDL groups at the time of PHx (Fig. 3B). As definitive histological changes are a lagging indicator for cholestatic injury, the apparent disconnect between plasma and histological injury profiles probably relates to the relatively short duration of combined OCA treatment and BDL.

rBDL rats adapted to BA overload by upregulating basolateral BA exporter *Mrp3* (Fig. 3C), which is analogous to the response seen in cholestatic mice and patients^28,29^. OCA treatment led to a more robust induction of *Mrp3* than in untreated rBDL rats, and concurrently upregulated basolateral bile salt exporters *Mrp4 and Ostβ* (Fig. 3C). An crucial role for *Mrp3* in mediating proliferation after PHx has been previously described under non-cholestatic conditions^30^. In addition, the archetypal Fxr target *Bsep*, coding for the canalicular BA exporter, was upregulated by ~8-fold (Fig. 3C).

**Figure 4:**
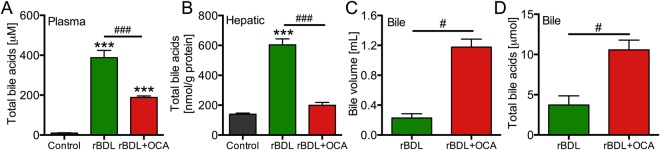
Obeticholic acid increases biliary bile acid output in rats with obstructive cholestasis. Shown are total bile acid (BA) levels in plasma (**A**) and liver tissue (**B**), and bile collected from the extrahepatic bile duct before decompression and subsequent PHx (**C**–**D**). The total BA data for the control group and the untreated BDL group were published previously^14^. Data were obtained after seven days of BDL (i.e., prior to PHx). Abbreviations: rBDL = reversible bile duct ligation; OCA = obeticholic acid. ***Indicates p < 0.001 versus the control group. ^#^Indicates p < 0.05 and ^###^indicates p < 0.001 between the experimental groups indicated by the solid line. *N* = 4–6 per group per time point.

As OCA induces choleresis^31^, the liver injury profile at baseline is likely caused by forced pumping of BAs via Bsep into the obstructed biliary tree. This can cause biliary infarcts due to heightened biliary pressure^27^. The increased volume of bile retrieved from the extrahepatic bile duct and elevated biliary BA output after seven days of OCA treatment support this notion (Fig. 4C,D). The marked induction of *Mdr2* (Fig. S7), which reduces biliary injury by reducing biliary BA toxicity, appeared inadequate to normalize liver biochemistry in rBDL rats treated with OCA. Comparable side effects of choleretic agents on BDL-induced liver injury have been reported previously^32^. A crucial role for Bsep in mediating this effect was substantiated by the positive correlation between *Bsep* expression, extrahepatic bile volume, and markers for biliary injury (Fig. S3) seen prior to PHx. Despite the reduction in hepatic BA content conferred by OCA (Fig. 4B), hepatocellular injury was also increased in OCA-treated animals at baseline. This could be secondary to the ductular reaction and related neutrophil influx (Fig. S6).

There were several findings that were difficult to reconcile. First, the basolateral BA importer *Ntcp* was slightly upregulated in the OCA group (Fig. 3C), whereas *Ntcp* is normally repressed by Fxr during cholestasis^14^. A similar loss of *Ntcp* suppression was previously seen in mice with intrahepatic (but not extrahepatic) cholestasis treated with the Fxr agonist GW4064^33^. It was also expected that hepatic *Cyp7a1* would be repressed by OCA^34–36^, either via direct of via Fxr-mediated induction of intestinal *Fgf15* (Fig. 1F). Despite evidence of hepatic OCA uptake (Fig. S8), consequent hepatocellular Fxr activation (discussed above) OCA did not alter hepatic *Shp* (Fig. 2D) or *Cyp7a1* mRNA levels (Fig. 3C). The rise in hepatic BA content caused by rBDL, however, was effectively counteracted by OCA treatment (Figs 4A,B and S9), which suggests that sustained BA production and uptake during OCA treatment likely has little harmful consequences.

#### Liver regeneration

Consistent with the restoration of bile flow, hepatobiliary injury markers normalized within 48 h after PHx in rBDL groups, albeit recovery was more gradual in OCA-treated animals (Fig. 3A). Despite a ∼4-fold decline, ALP levels remained elevated in the OCA group (Fig. 3A). Bilirubin clearance also lagged marginally in OCA-treated animals (Fig. 3A), which may either reflect impaired canalicular bilirubin export by Mrp2 and compensatory basolateral export by Mrp3 (Figs 3C and S7)^37^ or reflect the rise in longer-lived albumin-bound bilirubin typically seen under cholestatic conditions^38^. Histologically, the modest degree of hepatocellular necrosis seen in the rBDL groups prior to PHx gradually subsided during the regeneration phase (Fig. 3B), consistent with the downward trend in serum injury markers. Periportal fibrosis with a non-ductular etiology was observed in all control rats on day 5 after PHx, suggesting this is part of the normal regenerative response. A similar degree of periportal to septal fibrosis was seen in both rBDL groups, indicating that the increase in biliary injury markers in the OCA group prior to PHx did not exacerbate liver injury after PHx. Accordingly, there was no mortality during the regeneration phase.

## Discussion

Impaired liver regeneration remains a serious risk for patients with parenchymal liver pathology who undergo a major liver resection. Accordingly, it was explored whether the Fxr agonist OCA could improve liver regeneration in rats with obstructive cholestasis. Most importantly, OCA triggered the growth of cholestatic rat livers prior to PHx. A similar increase in liver size was observed in healthy mice fed a cholic acid-containing diet, but was not seen in *Fxr* knock-out animals^7^. This indicates that Fxr (agonists) can stimulate liver growth in absence of established mitogenic triggers such as PHx.

Although it is unclear how, BAs seem able to modulate liver size, as is supported by the increased size of humanized mouse livers with unrestrained BA synthesis and high circulating BA levels^34^. It is however questionable whether an expanded BA pool alone is sufficient to trigger hepatocyte proliferation, as BDL also increases systemic BA levels but does not induce liver growth, possibly due to the lack of intestinal Fxr stimulation. This interaction between cholestasis and OCA treatment (i.e., Fxr activation) is also evident from our findings that OCA did not affect dry liver mass in healthy, non-cholestatic animals (Fig. S4). The correction for hepatic water content also seems important when investigating the ‘hepatostat’, as gross liver mass does increase following murine BDL^39^, indicating that the increase in wet liver mass likely represents a direct consequence of inflammation (e.g. edema). Although both an increase in dry liver mass and increase hepatocyte proliferation were seen in OCA-treated rBDL animals, it remains to be experimentally validated whether this increase in liver mass also results in improved liver function. This is particularly essential given that discrepancies between liver size and liver function readouts have been reported in the context of portal vein embolization^40^.

As FXR is expressed both in liver and intestine, it is important to explore whether the effects of OCA on liver growth originate in the liver and/or the small intestine. Our transcript analyses point towards the small intestine, as the strong induction of intestinal Fxr target *Fgf15* was coupled to activation of the proliferative hepatic Fgfr4-Stat3 pathway^10^. The effect of OCA on the expression of indirect Fxr targets such as *Ntcp* and *Cyp7a1*, was less clear. The apparent importance of intestinal Fxr in driving liver growth is supported by studies stating that Fgf15 is indispensable for liver regeneration after PHx^9,10^, whereas the selective deletion of hepatocyte Fxr only slightly delays regeneration^41^. Defining the relative contribution of intestinal and hepatic Fxr to liver regeneration is also important considering the increased biliary injury caused by OCA during rBDL, as is discussed below. The recently developed FGF19 analog NGM282 may circumvent apparent disadvantages. NGM282 reduces cholestatic liver injury in *Mdr2*^−/−^ and in BDL mice^42,43^ and suppresses BA synthesis in healthy volunteers^43^. More importantly, the FGF19 variant Fibapo promoted the regeneration of steatotic livers after PHx in diabetic *db/db* mice^44^. In addition to the FXR-FGF15/19 pathway, several other targets have been recently identified that could be pharmacologically exploited to augment liver regeneration, including the bile salt receptor TGR5 (G protein-coupled bile acid receptor 1) and constitutive androstane receptor^8,45,46^.

The current experiments cannot determine whether OCA is also able to accelerate liver regeneration when given after PHx, as post-PHx liver mass recovery likely is influenced by pre-resection hepatomegaly in the OCA group. The differences in liver size^26^ and downregulation of *Fgfr4* may blunt the proliferative response and may direct the mode of regeneration towards hypertrophy instead of proliferation^47^, thereby preventing a reliable comparison with regeneration dynamics in the other groups. In light of clinical translation, the ability to upsize the liver before surgery is more appealing than accelerating the growth of a small liver remnant.

There are several issues that need to be addressed to understand how OCA affects liver regeneration. First, PHx and biliary drainage were performed simultaneously in this model, whereas patients with perihilar cholangiocarcinoma generally undergo biliary drainage several weeks prior to surgery. The increase in biliary injury seen in the rBDL + OCA group, which has also been observed with other choleretic agents^32^, likely causes little harm in the setting of pre-operative biliary drainage. This premise is corroborated by the rapid normalization of injury markers during the regeneration phase and the therapeutic success of OCA in treating patients with primary biliary cholangitis^12^. Considering the induction of BA exporters, OCA could expedite BA clearance following biliary drainage. OCA might therefore reduce drainage-associated complications such as cholangitis by narrowing the interval between drainage and surgery. The notion that OCA and other Fxr agonists also augment biliary innate immune function^48^ and limit bacterial translocation in the gut^49^ could further help to suppress drainage-related complications.

Second, OCA has thus far only been used to treat patients with benign liver disorders^12,50,51^. Most patients who would benefit from the effects of OCA on liver growth, however, have hepatobiliary cancer. Proliferative interventions may induce tumor progression, as was demonstrated earlier in the context of PVE^52^. A potential effect of OCA on malignancies may differ per cancer type. On the one hand, the FXR target FGF19 could expedite the growth of FGFR4-positive hepatocellular carcinomas^53^. On the other hand, FXR suppresses the growth of perihilar cholangiocarcinoma^54^ and colorectal cancer^55^.

In conclusion, the current work shows that OCA triggers growth in cholestatic livers, but can exacerbate biliary injury when used during complete biliary obstruction. These findings highlight that FXR is a complex target for intervention under dynamic metabolic conditions influenced by cholestasis, biliary drainage, and partial liver resection. Additional OCA dose testing, timing, and safety studies in animal models better reflecting the target patient population will help to discern whether OCA is beneficial in the work-up of (post)cholestatic patients scheduled for major liver surgery.

## Electronic supplementary material

Supplementary Information
