# Supplementary material for: FXR agonist obeticholic acid induces liver growth but exacerbates biliary injury in rats with obstructive cholestasis
Source: Sci Rep. 2018 Nov 8;8:16529. doi: 10.1038/s41598-018-33070-1 (PMC6224438; doi:10.1038/s41598-018-33070-1)
Supplement: Supplementary file 1 — Supplementary Information [file 41598_2018_33070_MOESM1_ESM.docx]

**SUPPLEMENTAL INFORMATION**

**FXR agonist obeticholic acid induces liver growth but exacerbates biliary injury in rats with obstructive cholestasis**

Rowan F. van Golen^1#^, Pim B. Olthof^1#^, Daniël A. Lionarons^1,2,3#^, Megan Reiniers^1^, Lindy K. Alles^1^, Zehra Uz^1^, Lianne de Haan^1^, Bulent Ergin^1^, Dirk R. de Waart^2^, Adrie Maas^1^, Joanne Verheij^4^, Peter L. Jansen^5^, Steven W. Olde Damink^5^, Frank G. Schaap^5^, Thomas M. van Gulik^1^, Michal Heger^1*^

*1. Department of Experimental Surgery, Academic Medical Center, University of Amsterdam, Amsterdam, the Netherlands*

*2. Tytgat Institute for Liver and Intestinal Research, Academic Medical Center, University of Amsterdam, Amsterdam, the Netherlands*

*3. Oncogene Biology Laboratory, The Francis Crick Institute and University College London, London, United Kingdom*

*4. Department of Pathology, Academic Medical Center, University of Amsterdam, Amsterdam, the Netherlands*

*5. Department of Surgery, NUTRIM School of Nutrition and Translational Research in Metabolism, Maastricht University, Maastricht, the Netherlands*

# these authors contributed equally to this manuscript

*Corresponding author: Michal Heger

Department of Experimental Surgery

Academic Medical Center

University of Amsterdam

Meibergdreef 9

1105 AZ Amsterdam

the Netherlands

Tel: +31 20 5665573

Fax: +31 20 6976621

Email: m.heger@amc.uva.nl

**Table S1.** Histological scoring system

| **Confluent necrosis** | |  | **Ductular reaction** | |  |
| --- | --- | --- | --- | --- | --- |
|  | *Absent* | *0* |  | *Absent* | 0 |
|  | *Present* | *1* |  | *Periportal* | 1 |
|  |  |  |  | *Septal* | 2 |
|  |  |  |  | *Cirrhosis* | 3 |
| **Fibrosis** | |  |  | |  |
|  | *Absent* | 0 |  |  |  |
|  | *(Peri)portal inflammation* | 1 |  |  |  |
|  | *Septal/bridging* | 2 |  |  |  |
|  | *Cirrhosis* | 3 |  |  |  |

**Supplemental Table S2.** qRT-PCR primers

| **Target** | **Forward (5’ 🡪 3’)** | **Reverse (5’ 🡪 3’)** |
| --- | --- | --- |
| *Cyp7a1* | GCAGCCTCTGAAGAAGTGAGTGG | GATGCTGTCTAGTACCGGCAGG |
| *Ntcp* | TGAACCTCAGCATCGTGATGACC | GGACGATCCCTATGGTGCAAGG |
| *Mrp3* | ATGCTGGCCAAAATGCGGTTGC | CCAGGAGCCCTTGCAGTATTCC |
| *Mrp4* | GTTCTGGCAAAGACCTTGGATGC | CCGTGTATTCAATCACCCTCTCC |
| *Bsep* | CTCTGCTTTGCCTTTTCCCAGG | AGAGACCACCCTGAAAACGTGG |
| *Mrp2* | GAGTCTGAGGATGAATCTCGACC | TGCCCTATGCTCAGGTTGTCACC |
| *Fxr* | GTCATCCTCTCTCCAGACAGACA | GGTTGAATGTCCGGAGTTCTGTC |
| *Shp* | GCTAGAGGAACCCAACAGTGGT | CCTGGCACATCTGGGTTGAAGA |
| *Ostβ* | TGGGTCCCTGCTGTTGCGAG | CGTCTCTGGGCCTGGATCTGG |
| *B2m* | CCACCGGAGAATGGGAAGCCC | TCTCGGTCCCAGGTGACGGT |
| Ubc | ACACCAAGAAGGTCAAACAGGAAGA | AGACACCTCCCCATCAAACCCA |
| *Foxm1* | AGGCGCCCTCAAGAGCATCA | TGGTGCCAACACTTCCAGCCT |
| *Ccnd1* | AGTGTGACCCGGACTGCCTC | CCTCGGTGGCCTTGGGATCG |
| *Fgfr4* | AGCTCCAGGCGGGTGAGTGT | CGCTGACCACCTTCCTGGCT |
| *Fgf15* | AGGGCCAGAAACCTTCAAAC | GATCCATGCTGTCGCTCTC |
| *Cyp8b1* | CCTGAAGGGAATGCGGGCCA | TGGGGCCAAAGGAGAGGGGA |
| *Stat3* | CCGGCAAGGGCTTCTCGTTCTG | CCCGGGGGCTTTGTGCTTAGGAT |
| *Socs3* | GGGGCCCCTTCCTTTTCTTTACCA | GGCCCCCTCTGACCCTTTCTTTG |
| *Tgfβ* | ATACAGGGCTTTCGCTTCAGTGCT | CCCGGGTTGTGTTGGTTGTAGAG |
| *Hprt* | CCTCAGTCCCAGCGTCGTGATTA | TCAGCACACAGAGGGCCACA |
| *Mdr2 (abcb4)* | AGCCGTTTGGGCAAAGATACTC | CTGCTCCTCTAGCATTGGGGAA |
| *Klb* | GATGCCTACACGACCCGACG | ACACCGCTAAACTCCGGCTTA |
| *Cdc25b* | CCTCATTCCAGCTCTGCCCG | CCGGGCCTTGGGTTCTTCAA |


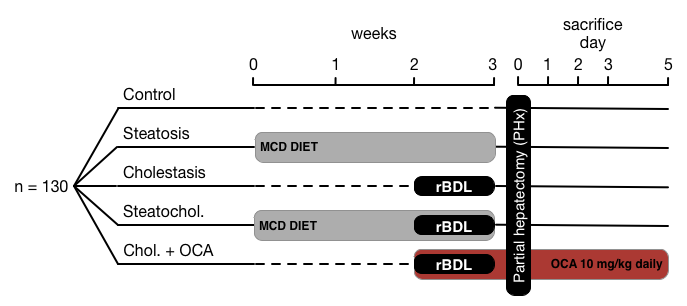


**Figure S1.** Schematic overview of the study design.

One hundred thirty rats were divided over five study arms. Animals were sacrificed before partial hepatectomy (PHx, i.e., sacrifice on day 0) or 1-5 days after PHx (n=4-6/group). In the rBDL groups, the enterohepatic circulation was restored directly before PHx by internal biliary drainage. Please note that based on the liver regeneration curves (Supplemental Figure S2), both simple steatosis (i.e., MCD diet) groups were omitted from detailed analysis and are therefore not elaborately addressed in the main text. rBDL = reversible bile duct ligation; Chol. = cholestasis; MCD = methionine- and choline-deficient diet; OCA = obeticholic acid; rBDL = reversible bile duct ligation; Steatochol. = combined simple steatosis and cholestasis.


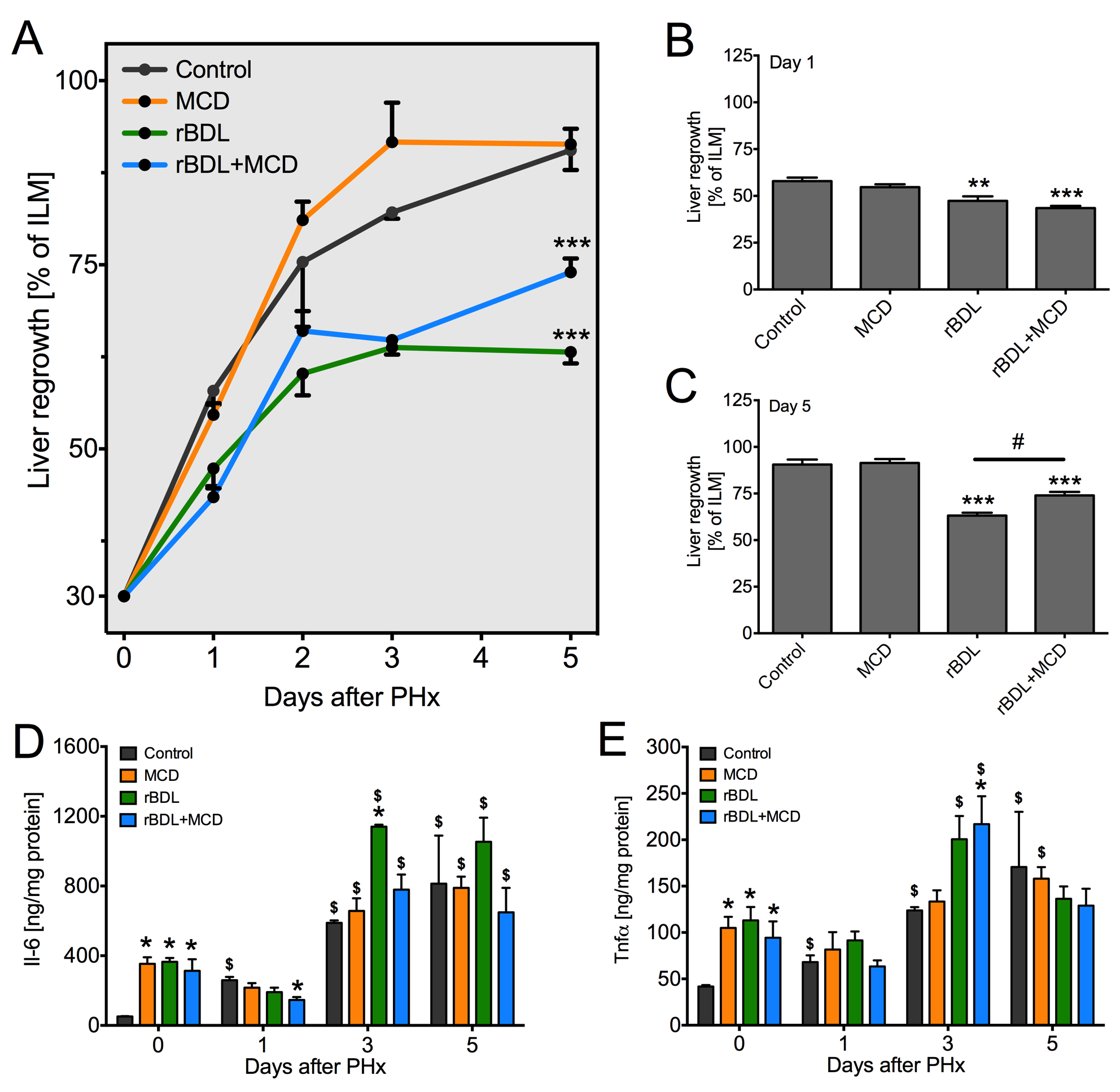


**Figure S2. Effects of parenchymal liver pathology on regeneration following partial hepatectomy**

**A** shows the liver regrowth in rats with healthy livers (black line) or hepatopathology (colored lines, see in-figure legend) during the first five days after 70% hepatectomy (PHx). Differences in liver regrowth on day 1 and day 5 post-PHx are also shown in **B** and **C**. Liver regrowth is expressed as the percentage of the initial (pre-PHx) liver mass (ILM). **D** and **E** depict hepatic levels of Tnfα and Il-6. All data are shown as mean±SEM of 4-6 rats per group per time point. Abbreviations: ILM = initial liver mass; MCD = methionine- and choline-deficient diet (simple steatosis); rBDL = reversible bile duct ligation (cholestasis). * indicates p < 0.05, ** indicates p <0.01, and *** indicates p < 0.001, all versus the control group. ^#^ signifies p < 0.05 between the experimental groups indicated by the solid line (panel **C**). ^$^ indicates p < 0.05 versus baseline (t = 0 days) within an experimental group.

**Materials and Methods Figure S2.**

A Diax 900 tissue homogenizer (Heidolph, Schwabach, Germany) was used to homogenize ±100 mg of rat liver on ice in 1400 μL of 5 mM NaPi buffer (pH=7.4) containing a protease inhibitor cocktail (cOmplete ULTRA, Roche). The homogenate was centrifuged for 10 min at 10,000×g (4^o^C) and Tnfα and Il-6 levels in the supernatant were measured by ELISA (R&D Systems, Minneapolis, MN) and normalized to homogenate protein content (Protein Assay Kit, Pierce, Rockford, IL).

**Results and Discussion Figure S2.**

The effects of rBDL and MCD treatment on parenchymal inflammation prior to PHx were reported previously^1^. Figure S2 shows that healthy rat livers regenerate to approximately 90% of their original mass in five days after PHx (Figure S2, black curve). Rat livers with steatosis regenerated as effectively as undamaged livers (Figure S2, orange curve), which corroborates earlier findings that steatohepatitis rather than the more prevalent simple steatosis induced by this short-term MCD diet compromises hepatic regeneration^2^. In contrast, liver regrowth was markedly impaired in rBDL animals with and without steatosis (Figure S2A-C). Impaired liver regeneration in these groups was already manifest one day after PHx (Figure S2A-B). The growth of rBDL livers stagnated on day 2 after PHx and did not exceed 60% of the initial liver mass, which was less than in the other study arms (Figure S2A and C). Please note that the regeneration curves for the control and rBDL groups are also shown in the main text (Figure 2A).

Pro-inflammatory cytokines play a key role in the initiation of liver regeneration after PHx^3^. Hepatic levels of the cytokines Il-6 and Tnfα were therefore quantified to determine whether the diverging regrowth rates were related to post-PHx cytokine production. Inherent to pre-existent inflammation^1^, hepatic cytokine levels were already elevated prior to PHx in all animals with parenchymal liver disease (Figure S2D-E). Il-6 and Tnfα increased on day 1 after PHx in healthy rats compared to baseline, whereas this increase was not observed in the other study groups (Figure S2D-E).


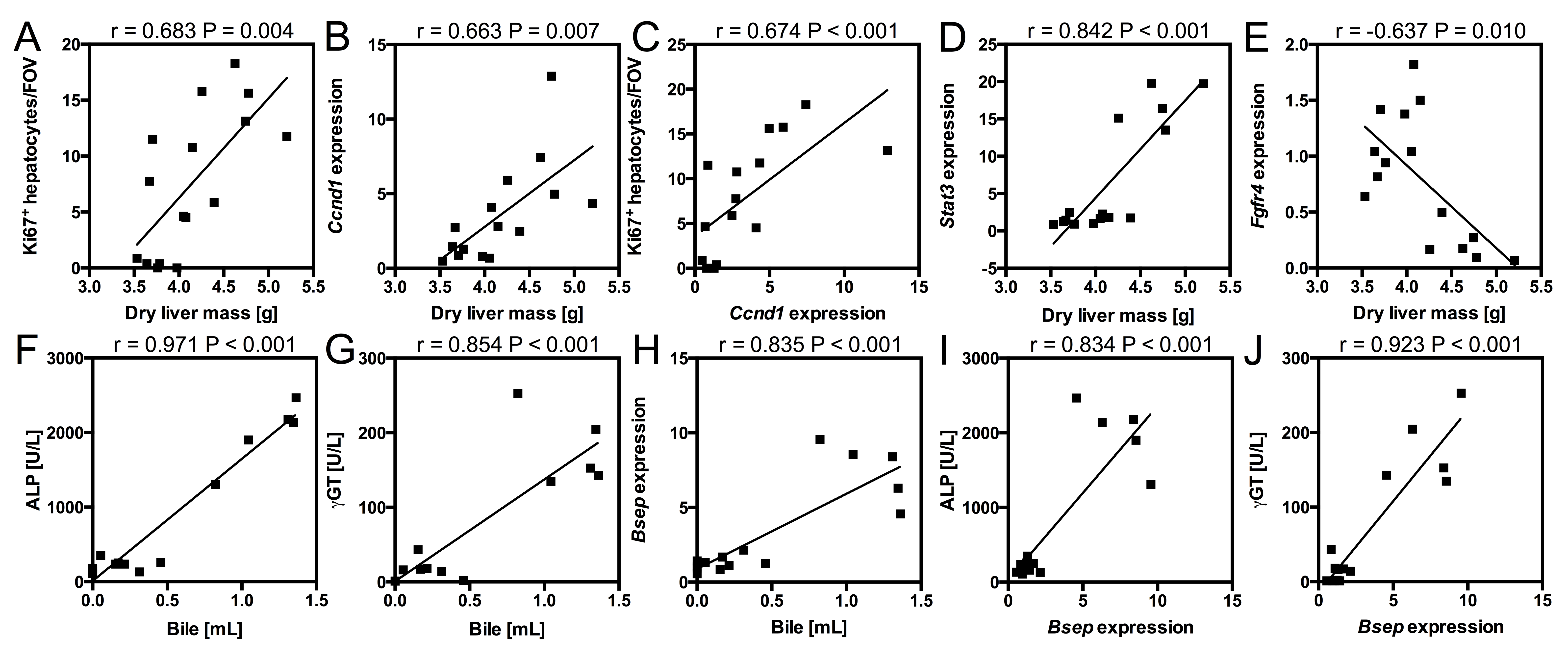


**Figure S3.** **Correlation analysis of liver growth and hepatobiliary injury parameters.**

**A-D** show positive correlations between liver weight, the number of Ki67-positive hepatocyte nuclei, and transcript levels of liver regeneration markers *Stat3* and *Ccnd1 (*encoding cyclin D1*)*. **E** indicates that an inverse relation exists between hepatic mRNA levels of the FGF15 receptor *Fgfr4* and liver weight, which supports the hypothesis raised in the main text (sections 3.2 and 3.3) that hepatocyte FGF15 signaling is controlled via a negative feedback loop (i.e., *Fgfr4* expression is suppressed in large livers). **F-J** show the association between the volume of bile retrieved from the extrahepatic bile ducts (*x*-axis, **F-H**), the expression of the canalicular bile acid (BA) transporter *Bsep* (*x*-axis, **I-J**), and markers for cholestatic injury (*y*-axis, **F-J**). These data support the premise that the increase in biliary injury observed in cholestatic rats on obeticholic acid treatment prior to liver resection is caused by induction of BSEP, leading to forced BA export into an obstructed bile duct. These findings are discussed in detail in section 3.4.1 of the main manuscript. All correlations were tested using Spearman’s correlation coefficient (n=4-6/group). ALP = alkaline phosphatase; FOV = field of view; γGT = gamma-glutamyl transferase.


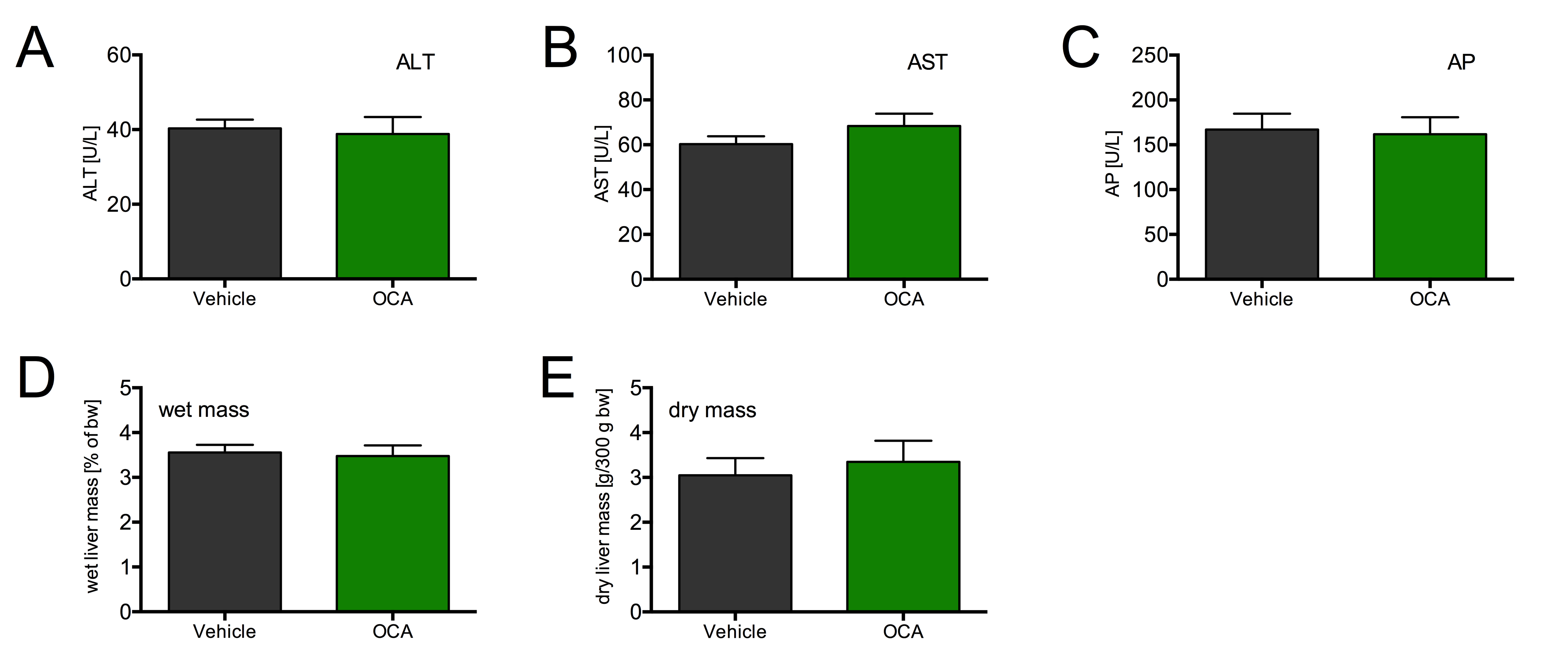


**Figure S4. Seven days of obeticholic acid treatment is not toxic and does not induce liver growth in healthy rats.**

Shown are plasma markers for hepatocellular injury (**A-C**) and liver mass parameters (**D-E**) obtained from healthy (i.e., non-cholestatic) rats, treated daily with either 10 mg/kg of obeticholic acid (OCA) per oral gavage or vehicle only (1% methylcellulose) per oral gavage. Animals were sacrificed after seven days of OCA or vehicle treatment (i.e., no hepatctomy was performed). In the main text, it was shown that OCA treatment in cholestatic (rBDL) rats leads to liver growth, but also exacerbates biliary injury.The experiments shown here served to expore whether a similar effect was seen in healthy, non-cholestatic rats. As is evident from the top row, neither OCA nor vehicle treatment affected plasma transaminase levels (**A-B**) or levels of alkaline phosphatase. Bilirubin and gamma glutamyltransferase were not detectable in the plasma of either group (data not shown), reaffirming that OCA was non-toxic under these conditions. In contrast to the data shown in the main text for cholestatic (i.e., rBDL) animals, OCA did not affect wet (**D**) or dry (**E**) liver mass (discussed in the text). Data are shown as mean±SD of 6 animals per group. All experimental protocol were as described in the materials and methods section of the main text.


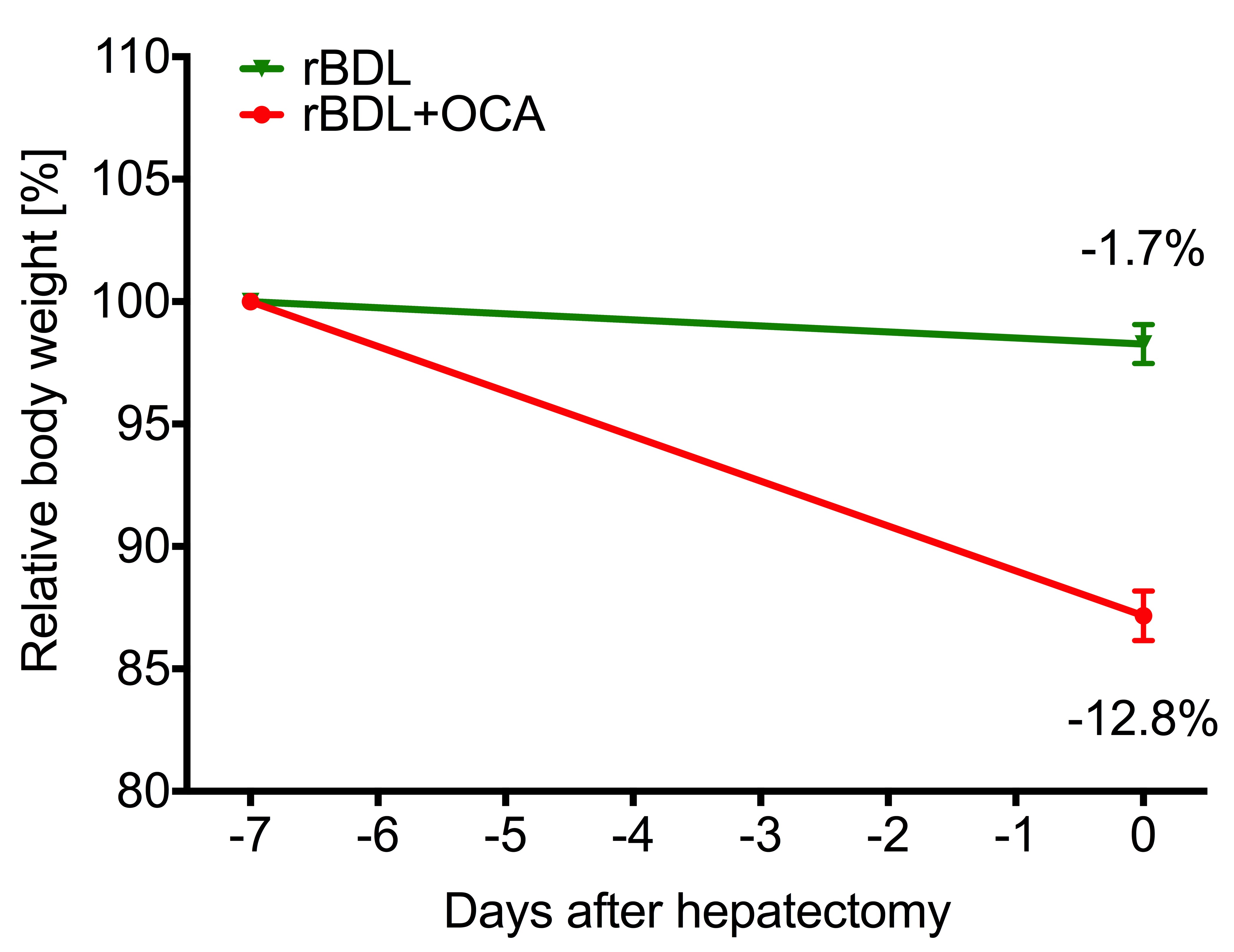


**Figure S5.** **Body weight dynamics.**

Shown are the changes in rat body weight in the cholestasis (rBDL) and rBDL+OCA groups, measured at baseline (t=-7) and after seven days of BDL (i.e., on the day of partial hepatectomy, t=0). It is shown that body weight decreased by almost 2% in the rBDL group, whereas a more substantial weight loss of ~13% was seen in the rBDL+OCA groups. Data are shown as mean±SEM of 16-31 animals per study group.


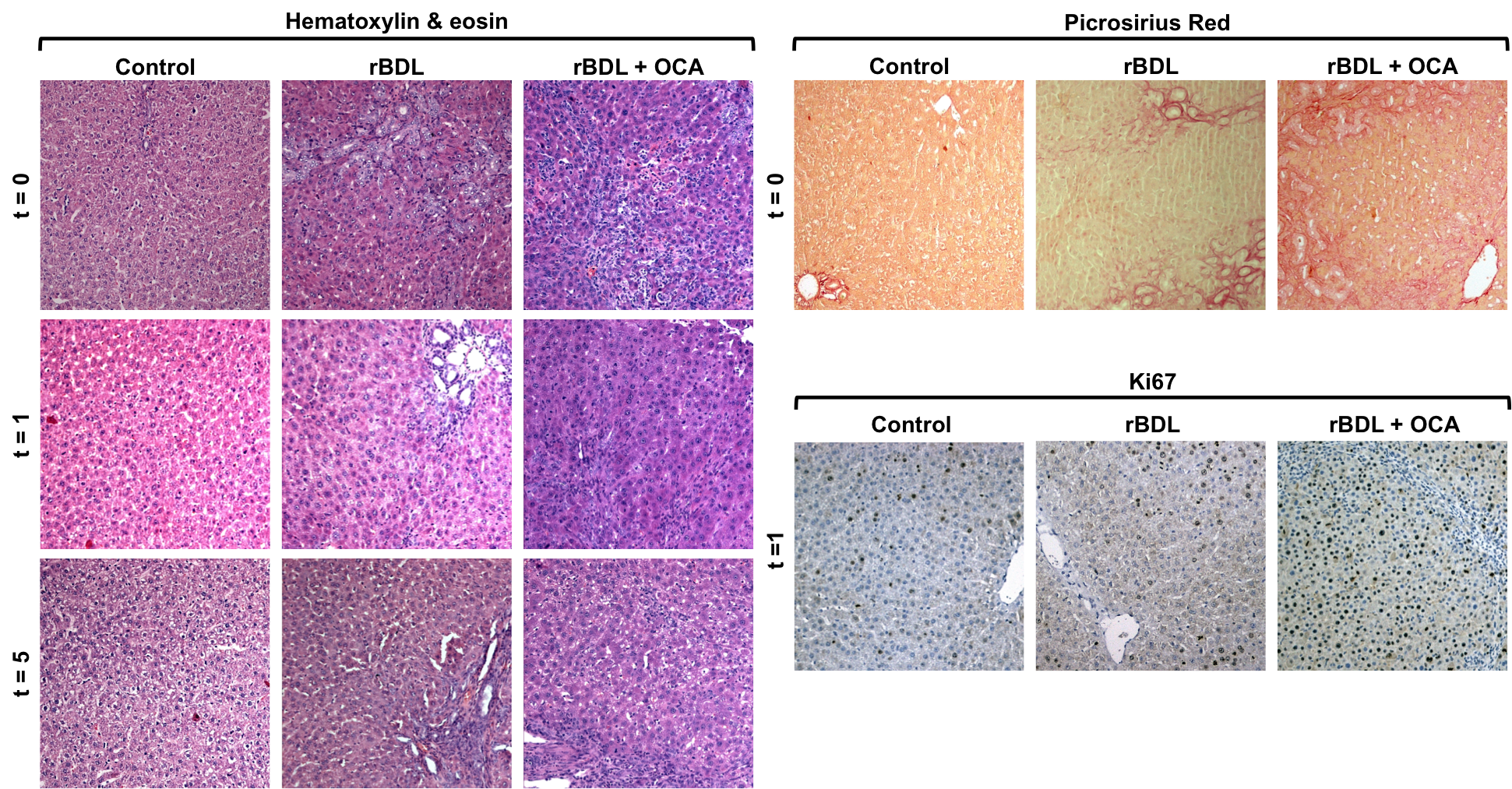


**Figure S6. Liver histology**

Shown are representative images of hematoxylin and eosin- (H&E, left), picrosirius red (top right), and Ki67-stained liver slides. All images were acquired with a 10× objective. The study groups (columns) are included above the panels and the time point at which the liver sections were obtained are shown on the left (rows). The H&E slides show inflammatory changes in the cholestasis and obeticholic acid (OCA) group prior to partial hepatectomy (i.e., t=0), which gradually resolve during the regeneration phase (t=1 and t=5). Picrosirius red-stained histology shows the onset of bridging fibrosis in cholestatic animals with and without OCA treatment prior to PHx (t=0). The Ki67-stained slides show the early peak in hepatocyte proliferation (i.e., number of Ki67-positive hepatocyte nuclei) on day one after PHx in OCA-treated animals. All liver histology was semi-quantitatively scored using the scoring system detailed in Supplemental Table S1. Results of the histological assessment are shown in Figure 5 of the main text and are discussed in more detail in section 3.4. Chol. = cholestasis; OCA = obeticholic acid.


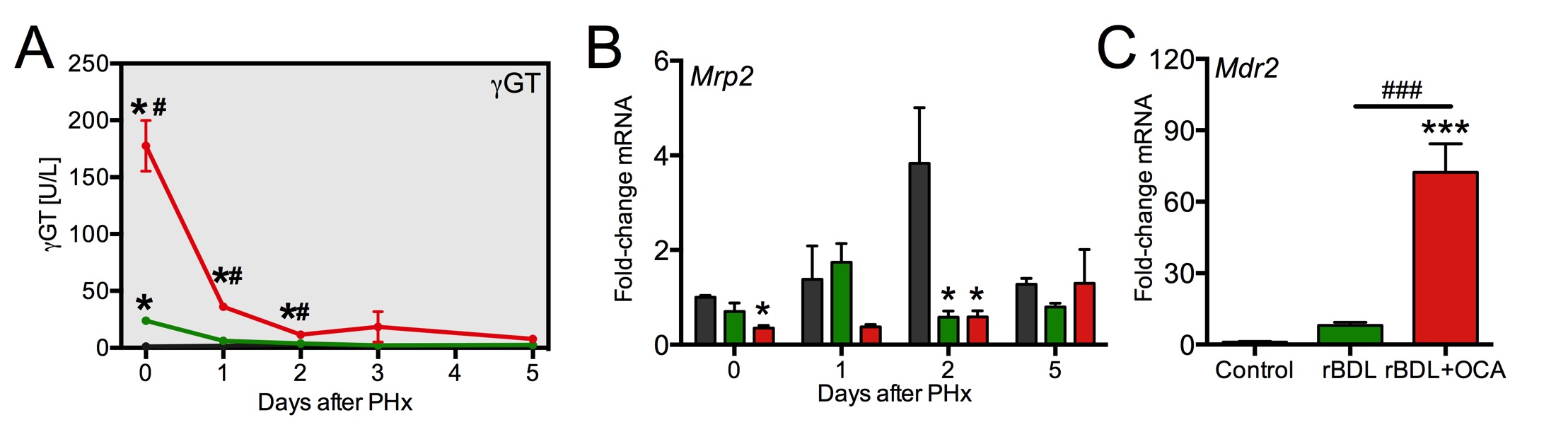


**Figure S7 – Biliary injury and changes in hepatic transporter expression in rats with obstructive cholestasis before and after partial hepatectomy.**

**A** shows the dynamics of the biliary injury marker gamma glutamyltransferase (γGT) prior to partial hepatectomy (PHx, day 0) and after PHx with concurrent biliary decompression. As is shown for alkaline phosphatase in the main text (Figure 5B), γGT was substantially elevated prior to PHx (day 0). In contrast to ALP, γGT levels normalized within 3 days following PHx. **B** and **C** show hepatic mRNA levels of the canalicular exporters *Mrp2* and *Mdr2*. *Mdr2* levels are only shown at baseline (prior to PHx, day 0).


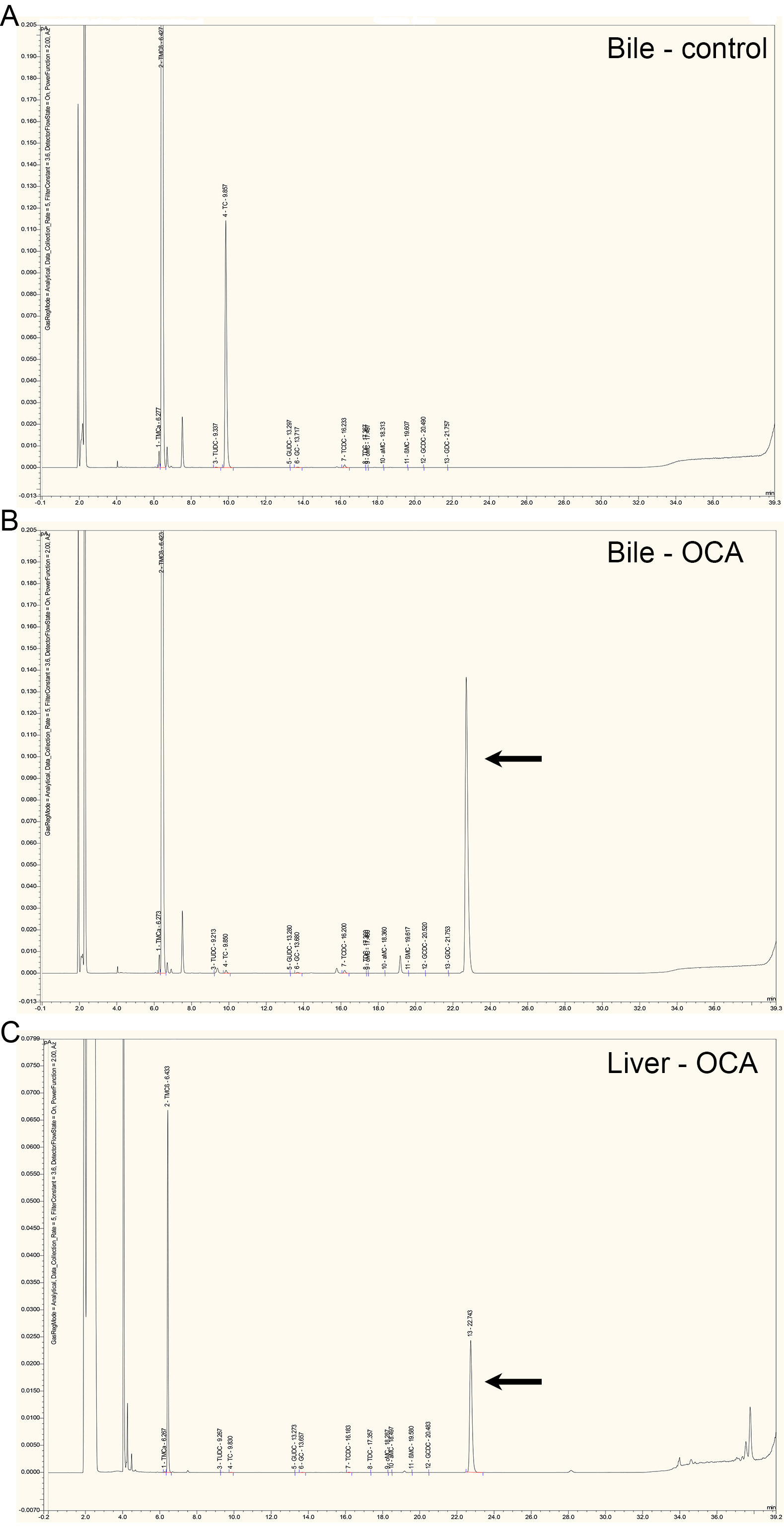


**Figure S8. Obeticholic acid uptake and secretion in cholestatic rat livers.**

Shown are representative high-performance liquid chromatography (HLPC) chromatograms of the bile acid (BA) pool composition in bile (**A,B**) or liver (**C**) samples obtained from rats rendered cholestatic by bile duct ligation. The relative abundance of each BA is reflected by the absorbance in arbitrary units (y-axis), which is plotted as a function of retention time in minutes (x-axis). Samples were obtained after 7 days of BDL. The arrow in **B** indicates the presence of an additional species with a retention time of just under 23 min in the bile of rats fed OCA, which was not seen in control animals (**A**). A similar peak was observed in the livers of OCA-exposed rats (**C,** arrow). Although no standards were available for reference, this additional peak most likely represents glycine- or taurine-conjugated OCA. These data therefore provide circumstantial for the uptake, conjugation, and biliary secretion of OCA in BDL rats. Experimental protocols are described in the Materials and Methods section of the main text. α/β/ω/MC = alpha/beta/omega muricholic acid; TC = taurocholic acid; TDC = taurodeoxycholic acid; TCDC = taurochenodeoxycholic acid; TUDC = tauroursodeoxycholic acid; Tα/βMC = tauro alpha/beta muricholic acid; GC = glycocholic acid; GCDC = glycochenodeoxycholic acid; GUDC = glycoursodeoxycholic acid.


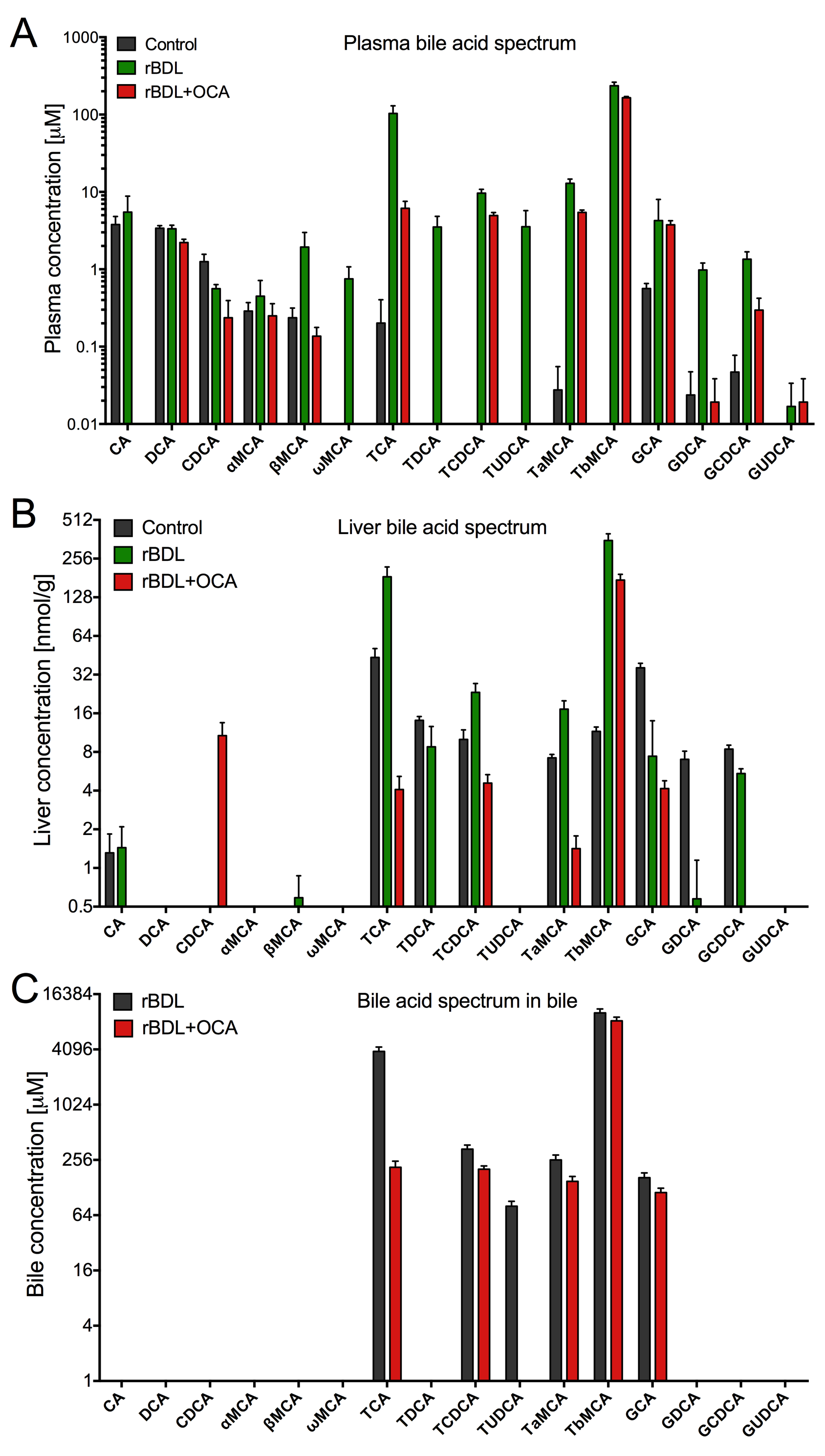


**Figure S9. The influence of obstructive cholestasis and obeticholic acid treatment on the bile acid pool composition of plasma, liver, and bile in rats.**

Shown are bile acid spectra measured in plasma **(A)**, liver **(B)**, and bile **(C)** samples of control rats (black bars), BDL rats (green bars), or BDL rats treated with the Fxr agonist obeticholic acid (OCA) for seven days (red bars). All samples were obtained at baseline (i.e., t=0, prior to hepatectomy). Note that bile samples were not available from non-cholestatic control animals. Bile acid profiles were determined by HPLC as described in the materials and methods section of the main manuscript. Data are shown as mean±SEM of 5-6 samples per group. The *y*-axis is scaled logarithmically to increase figure legibility. The plasma and hepatic bile acid pool composition in the rBDL and control groups were published previously^1^. CA = cholic acid; DCA = deoxycholic acid; CDCA = chenodeoxycholic acid; α/β/ω/MCA = alpha/beta/omega muricholic acid; TCA = taurocholic acid; TDCA = taurodeoxycholic acid; TCDCA = taurochenodeoxycholic acid; TUDCA = tauroursodeoxycholic acid; Tα/βMCA = tauro alpha/beta muricholic acid; GCA = glycocholic acid; GDCA = glycodeoxycholic acid; GCDCA = glycochenodeoxycholic acid; GUDCA = glycoursodeoxycholic acid.

**Figure S10. Liver function parameters are similar between cholestatic and control rats before and after partial hepatectomy.**


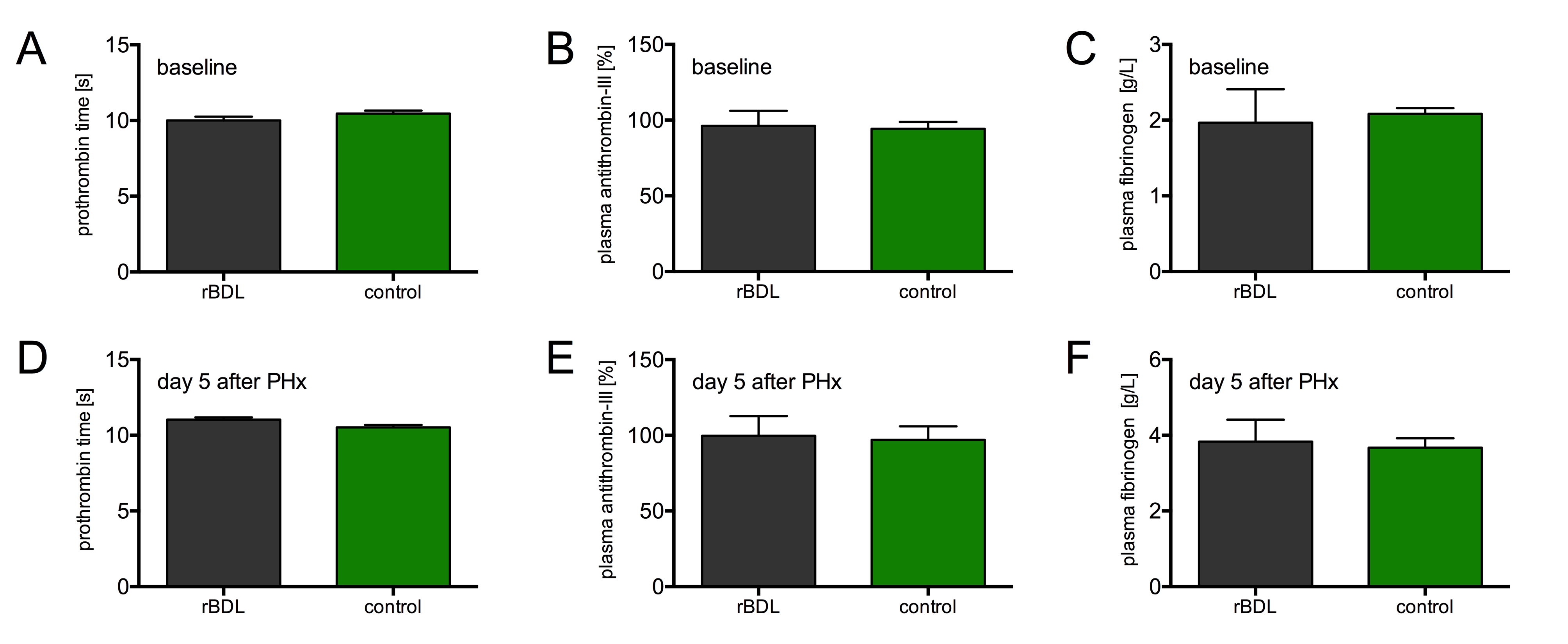


Shown are the liver function parameters prothrombin time (A+D), anithrombin-III activity (B+E), and fibrinogen levels (C+F), as measured by routine clinical chemistry. Rats were exposed to seven days of reversible bile duct ligation (rBDL, black columns) or underwent a sham operation (control, green bars). After seven days, all animals underwent a partial hepatectomy (70%, PHx), during which cholestasis was relieved through internal biliary drainage (see Materials and Methods, main text). Blood samples were obtained seven days after rBDL or sham surgery (‘baseline’, top row) or five days after PHx. There were no intergroup differences in liver function parameters. Data are shown as mean±SD of 4-6 animals per time point. PHx = partial hepatectomy.

**Supplemental Materials and Methods – Power Calculation**

The study was powered on the difference in liver regeneration between cholestatic (rBDL) animals and healthy (control) animals on day 5 after partial hepatectomy (Fig. S2). Based on a difference between means of 27.4, standard deviations of 3.1 and 5.4, and a required power of ≥0.80, group sizes of three animals were calculated. For the obeticholic acid experiments, the estimated effect size was set at 50% and the anticipated standard deviation increased to 6.5 to account for the increasing complexity of the experiments, amounting to a minimum of 5 animals per group. One animal was added to each group to compensate for potential attrition.

**References**

1. Lionarons, D. A., Heger, M., van Golen, R. F., Alles, L. K., *et al.* Simple steatosis sensitizes cholestatic rats to liver injury and dysregulates bile salt synthesis and transport. *Scientific Reports* **6,** 31829 (2016).

2. Reddy, S. K., Marsh, J. W., Varley, P. R., Mock, B. K., *et al.* Underlying steatohepatitis, but not simple hepatic steatosis, increases morbidity after liver resection: a case-control study. *Hepatology* **56,** 2221-2230 (2012).

3. Taub, R. Liver regeneration: from myth to mechanism. *Nat Rev Mol Cell Biol* **5,** 836-847 (2004).
